# Design and Optimization of a Fan-Out Wafer-Level Packaging- Based Integrated Passive Device Structure for FMCW Radar Applications

**DOI:** 10.3390/mi15111311

**Published:** 2024-10-29

**Authors:** Jiajie Yang, Lixin Xu, Ke Yang

**Affiliations:** School of Mechatronical Engineering, Beijing Institute of Technology, Beijing 100081, China; yangjiajie@bit.edu.cn (J.Y.); 3120205167@bit.edu.cn (K.Y.)

**Keywords:** integrated passive device, fan-out wafer level package, electromagnetic simulation, surrogate model, optimization

## Abstract

This paper presents an integrated passive device (IPD) structure based on fan-out wafer-level packaging (FOWLP) for the front end of frequency-modulated continuous wave (FMCW) radar systems, focusing on enhancing the integration efficiency and performance of large passive components like antennas. Additionally, a new metric is introduced to assess this structure’s effect on the average noise figure in FMCW systems. Using this metric as a loss function, we apply the support vector machine (SVM) for electromagnetic simulation and the genetic algorithm (GA) for optimization. The sample fitting variance is 2.42 dB, reducing computation time from 12 min to under 1 millisecond, with the entire optimization completed in less than 100 s. The optimized IPD structure is 0.7 × 0.9 × 0.014 ${\lambda_{0}}^{3}$ in size and achieves over 35 dB isolation between the transmitter and receiver. Compared to the IPD model calculated by empirical formulas, the optimized device lowers the average noise figure by 15.2 dB and increases maximum gain by 4.19 dB.

## 1. Introduction

The X-band frequency-modulated continuous wave system (FMCW), with its exceptional high resolution, excellent penetration capability, and miniaturized design, has been widely applied in fields such as weather monitoring, geological surveys, target detection and tracking, and health monitoring [1,2,3]. As application scenarios and technological processes evolve, FMCW radar systems are progressively moving towards higher integration, lower power consumption, and enhanced reliability, thereby generating a significant demand for miniaturization [4,5,6,7,8]. The FMCW system typically consists of active chips and passive structures, with passive components, such as antennas and microstrip lines, occupying the majority of the system’s integrated area. Their size is closely related to the operating frequency band and substrate material parameters, which poses one of the main challenges for system miniaturization.

To achieve the system miniaturization with integrated antennas, Yueping Zhang proposed the concept of antenna-in-package (AiP) [9], which enables the integration of patch antennas with other components. According to the substrate materials, AiP systems can be categorized into ceramic-based [10,11], silicon-based [12,13], glass-based [14,15], and organic-based [16,17], covering frequency from the X-band to THz. Organic-based AiP systems provide lower dielectric constants, reduced costs, and simpler manufacturing processes than systems made from other materials. Technologies using organic substrates include printed circuit board (PCB), high density interconnect (HDI), and fan-out wafer-level packaging (FOWLP). FOWLP directly rearranges and encapsulates chips on the wafer, eliminating laminated boards and wire bonding, improving stability, and reducing parasitic effects. Several FOWLP-based AiP examples have been reported [17,18,19,20,21,22,23,24], mainly for antennas, antenna arrays, and backside ground layers, resulting in thinner substrates [19]. This paper uses FOWLP’s dual-sided multi-layer redistribution to vertically integrate antennas with passive microstrip devices, forming an integrated passive device (IPD) for X-band FMCW RF front ends.

Regarding the evaluation and optimization of microwave structures, the above-mentioned articles typically use finite element simulations to obtain indicators such as return loss, antenna gain, and the isolation between the transmitter and receiver. However, these studies often overlook the actual application of the structures during the evaluation and optimization process. This also turns the optimization of the microwave devices into a multi-objective optimization problem. For example, in [25], the weighting distribution in the multi-objective optimization of antenna return loss and gain has not been thoroughly discussed. Besides, the IPD proposed in this paper plays a key role in FMCW systems, where the receiver faces greater challenges and noise interference compared to the transmitter, including environmental noise through the antenna, transmitter leakage, and intrinsic noise from components [26,27,28,29]. Therefore, factors such as return loss, isolation, and efficiency must be considered alongside the overall performance of the FMCW system. To address the lack of practical significance in multi-objective optimization found in existing papers and the need for noise evaluation in FMCW receivers, we theoretically analyze how the return loss, isolation, and efficiency of the IPD structure affect the noise figure of the FMCW system. Additionally, we propose and optimize a single metric to represent the impact of IPD parameters on the system’s average noise.

This paper leverages the benefits of FOWLP technology to design an ultra-thin IPD structure for the FMCW RF front-end, while also proposing a metric that reflects the impact of this structure on the receiver’s average noise figure. A surrogate model based on support vector machine is employed, with genetic algorithm serving as the optimization method, and the proposed metric is used as the objective for optimizing the IPD structure. The remainder of this paper is organized as follows: Section 2 outlines the process flow and initial size design of the structure based on theoretical calculations. Section 3 details the establishment of the simulation model, evaluates the structural parameters, and derives the new evaluation metric. Section 4 applies support vector machine and genetic algorithms to optimize the structure dimensions, using the new evaluation metric as the objective function. Finally, Section 5 presents the conclusions.

## 2. Structure, Process, and Empirical Formula-Based Dimensioning of the Integrated Passive Device

### 2.1. Structure Design of the Integrated Passive Device

**Figure 1:**
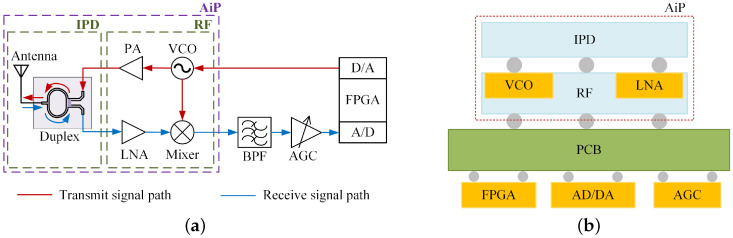
Schematic diagram of the FMCW detection system. (**a**) Block diagram. (**b**) Integrated architecture.

Figure 1a illustrates a typical FMCW short-range detection system, which is divided into a transmission link and a reception link. The baseband signal is output by a digital chip (FPGA) and converted from digital to analog (D/A) before being delivered to a voltage-controlled oscillator (VCO). The VCO outputs a modulated radio frequency signal that is transmitted through a power amplifier (PA) to the antenna, while another path enters a mixer as the local oscillator (LO) signal. The echo signal received by the antenna is amplified by a low-noise amplifier (LNA) before being fed into the mixer, where it is mixed with the LO signal. The mixed output then passes through a bandpass filter (BPF) for filtering, followed by an automatic gain controller (AGC) to produce a stable detection signal. Finally, this detection signal is converted into a digital signal via an analog-to-digital converter (A/D) and processed by the digital chip (FPGA). The transmitter and receiver in Figure 1a share one common antenna, with the duplexer utilized to isolate the signals.

Compared with the FMCW systems using other duplexers, the circuit structure of the system using Wilkinson power divider as the duplexer is simpler and more cost-effective. Similarly to the integration issue with microstrip antennas, the size of the power divider, composed of microstrip lines and isolation resistors, is also constrained by the RF frequency of the system. Therefore, we consider vertically integrating it with the antenna to form the integrated passive device (IPD) for the X-band FMCW system front end.

As shown in Figure 1b, this paper mainly discusses the integration and optimization of IPD, which is the first layer of the AiP structure. Other RF devices (VCO, Mixer, etc.) are located in the second layer of the AiP structure, and the solder ball is used to integrate the two layers. After integrating IPD and other RF dies by FOWLP technology into the AiP structure, they are soldered onto the PCB along with other chips (FPGA, AGC, etc.) to form the FMCW system.

**Figure 2:**
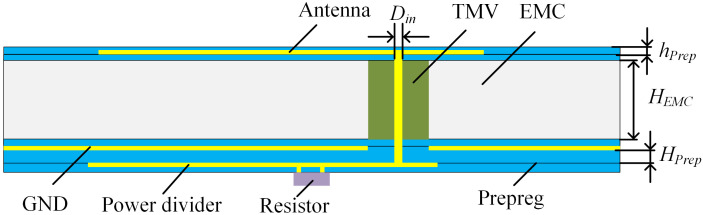
Side view of IPD based on FOWLP technology.

A coaxially feeding rectangular patch antenna is used, which has the advantages of simple structure and ease of fabrication. Figure 2 illustrates the IPD structure based on FOWLP technology, where through mold via (TMV) is integrated into the epoxy molding compound (EMC) substrate as a feedthrough via hole for the antenna. The antenna, ground layer, and the power divider are fabricated on both sides of the substrate using the redistribution layer technology, with the resistor positioned on the side of the power divider. The redistribution layers comprises multiple layers of copper (Cu) and the prepreg. The descriptions and values of the non-modifiable structural parameters used in the FOWLP process are summarized in Table 1.

### 2.2. Process for Fabricating the Integrated Passive Device Using Fan-Out Wafer Level Package Technology

**Figure 3:**
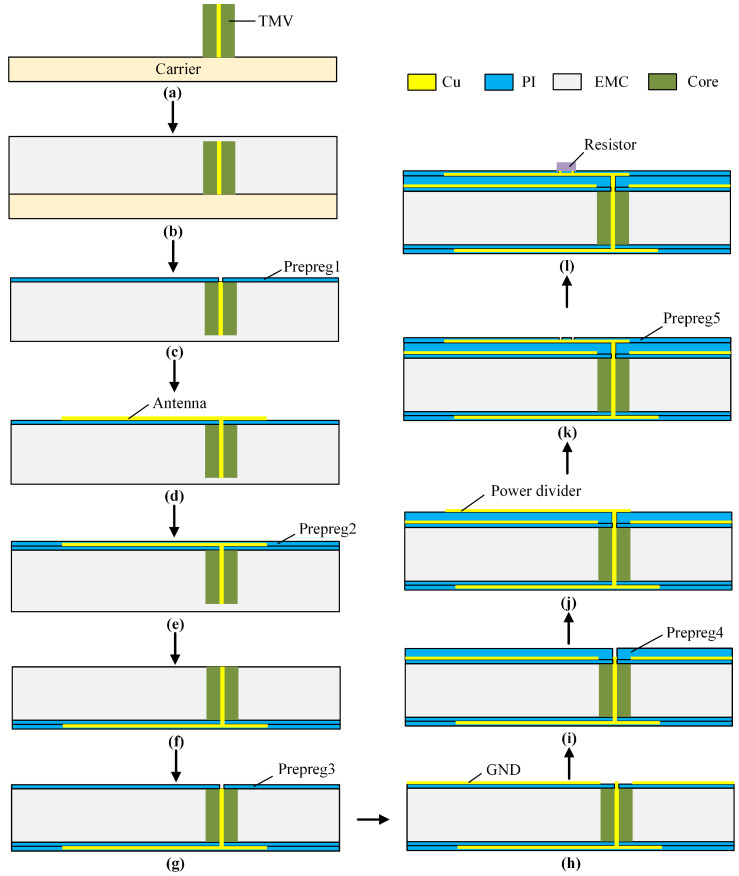
Process flow of the proposed IPD based on FOWLP technology. (**a**) Loading TMV on the carrier; (**b**) Injecting EMC as the substrate for the antenna; (**c**) Debonding and preparing the first prepreg layer; (**d**) Electroplating the antenna; (**e**) Preparing the second prepreg layer; (**f**) Thinning EMC to expose the TMV chip; (**g**) Preparing the third prepreg layer; (**h**) Electroplating the ground layer and feeding port; (**i**) Preparing the substrate for the power divider; (**j**) Electroplating the power divider; (**k**) Preparing the fifth prepreg layer; (**l**) Electroplating the pads and mounting the resistor.

The general process of FOWLP includes loading, injection, and formation of the redistribution layer (RDL) [17,18,19]. The process flow is shown in Figure 3.

Firstly, TMV is placed onto the carrier, and is temporarily bonded by the release layer, which is not shown in Figure 3a. Then EMC is injected to encapsulate the wafer, forming the substrate for the antenna. Next, the wafer is debonded from the carrier, exposing one side of the TMV. The first prepreg layer is then applied to the wafer and etched to create the feeding. Electroplating is used to deposit the first metal layer, which serves as the antenna, onto the first prepreg layer. The second prepreg layer is made to protect the finished structure. After that, the other side of the wafer is thinned to expose the TMV. Subsequently, the RDL process for the structure on the opposite side of the wafer is illustrated in Figure 3f–j, which involves preparing the ground layer and the power divider. It is important to note that the thickness of the fourth prepreg layer, serving as the substrate for the power divider, is greater than that of other prepreg layers. This is necessary to ensure the correct impedance values for the microstrips used in the power divider. Figure 3k,l demonstrate the process of mounting the isolated resistor on the top layer.

### 2.3. Empirical Formula-Based Dimension Design of the Integrated Passive Device

**Figure 4:**
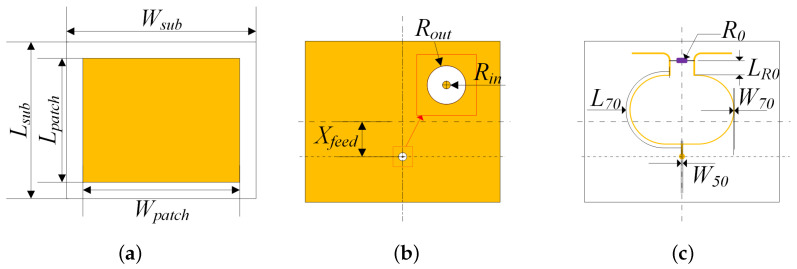
Top view of IPD layers. (**a**) Antenna layer. (**b**) GND layer. (**c**) The power divider layer.

The detailed layouts of IPD is shown in Figure 4, where $W_{sub}$ and $W_{patch}$ are the widths of the substrate and the antenna, $L_{sub}$ and $L_{patch}$ are the lengths of the substrate and the antenna, and $X_{feed}$ is the position of the feed point, $R_{out}$ refers to the outer radius of the ring between the signal via and the ground, $R_{in}$ is the radius of the through mold via, and the corresponding diameter values are already provided in Table 1. $W_{50}$ and $W_{70}$ are the widths of the microstrip lines with characteristic impedance values of 50 Ω and 70.7 Ω, $L_{70}$ is the length of the quarter-wavelength transmission line with impedance of 70.7 Ω, $R_{0}$ is the value of the isolation resistor, and $L_{R_{0}}$ is the relative length of the resistor to the interface of the quarter-wavelength transmission line, the value of which is generally zero in the theoretical design. The center frequency discussed in this article is 8.5 GHz.

We use the formulas in [30] to calculate the structural parameters for the patch antenna. The coaxial line model is used to calculate $R_{out}$, where the characteristic impedance is 50 Ω, inner radius is $R_{in}$, and the filler material is EMC. When designing the power divider, it is necessary to solve the microstrip line widths with characteristic impedance of 50 Ω and 70.7 Ω, as well as the length of the quarter-wavelength microstrip line. The values of the line widths can be calculated using the transmission line model in [31]. The parameters of IPD obtained through Empirical formula-based design are recorded in Table 2.

## 3. Evaluation and Analysis of Simulation Results for the Empirical Formula-Based Design

### 3.1. Simulation Results Corresponding to the Empirical Formula-Based Design

**Figure 5:**
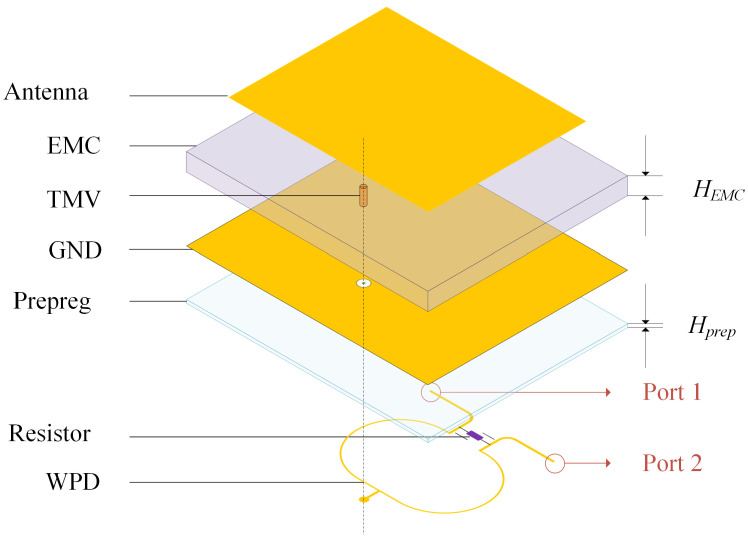
Simplified exploded view of IPD based on FOWLP technology.

We present a simulation model of the IPD structure based on the FOWLP process, as shown in Figure 5. This model retains only the primary dielectric layers that affect the antenna and Wilkinson power divider, while neglecting other process-related details for the purpose of calculation and simulation. In practical manufacturing, the thickness of the EMC is often several orders of magnitude greater than that of the prepreg material, as shown in Table 1. Additionally, the dielectric constants of EMC and prepreg are approximately similar. Therefore, in antenna simulations, the thickness of the prepreg not used as the component’s substrate, denoted as $h_{prep}$ in Table 1, can be considered negligible. However, for the power divider, the prepreg material acts as the dielectric medium for electromagnetic wave transmission between the power divider and the ground layer. Hence, the thickness of the prepreg between the power divider and the ground layer, denoted as $H_{prep}$ in Table 1, must be considered for accurate parameter calculations of the power divider. In Figure 5, Ports 1 and 2 are connected to the receiver and transmitter of the FMCW system, respectively.

**Figure 6:**
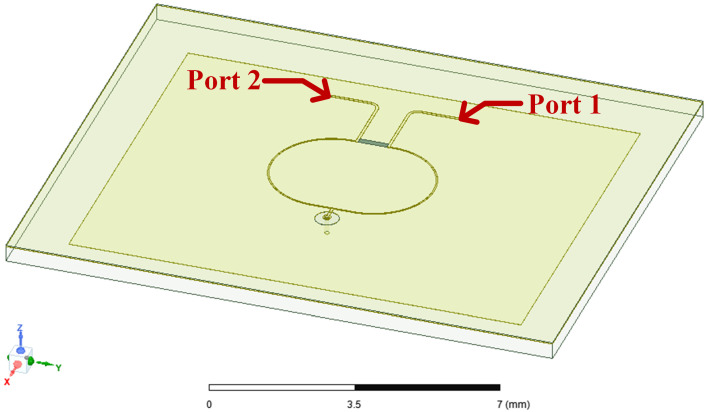
Model of the integrated passive device (IPD) in the finite element method (FEM) solver HFSS.

**Figure 7:**
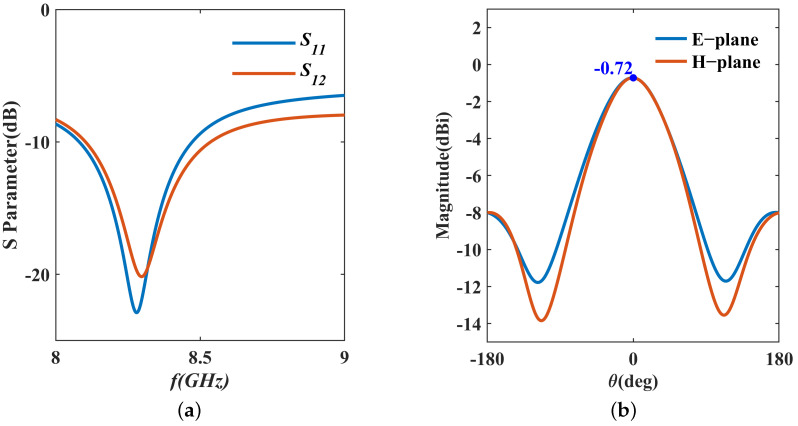
Simulation results of IPD designed by empirical formulas. (**a**) S-parameters. (**b**) E-plane and H-plane.

Figure 6 shows the model of the integrated passive device (IPD) in the finite element method (FEM) solver HFSS. Unlike the traditional power synthesis and splitting function of power dividers, we use only Port 2 as the feed port for the IPD structure, with Port 1 connected to a 50 Ω load for structure simulation. The material and structural parameters for simulation are based on the values in Table 1 and Table 2. The S-parameters of the IPD are illustrated in Figure 7a. From these results, we can observe a shift in the center frequency, with the $S_{11}$ value higher than −10 dB at 8.5 GHz, and $S_{12}$ higher than −15 dB. Additionally, Figure 7b displays the gains of the IPD, with a maximum value of only −0.72 dBi, which is not suitable for practical measurements.

The simulation results of the empirical formula-based design indicate that the model needs to consider multi-objective optimization from the perspectives of $S_{11}$, $S_{12}$, and the antenna efficiency, which present a complex challenge for the system. Given that the IPD structure serves both antenna and duplexer functions in the FMCW system, it plays a crucial role in the quality of the transmitted and received signals. Therefore, we aim to optimize the three parameters with a focus on signal quality within a specific frequency band. By integrating them into a single metric, we can effectively evaluate the performance of the IPD system, which will facilitate subsequent optimizations. This leads us to the proposed average noise figure metric, which will be discussed in the next subsection.

### 3.2. Modeling the Effect of IPD on the Receiver Noise Figure

**Figure 8:**
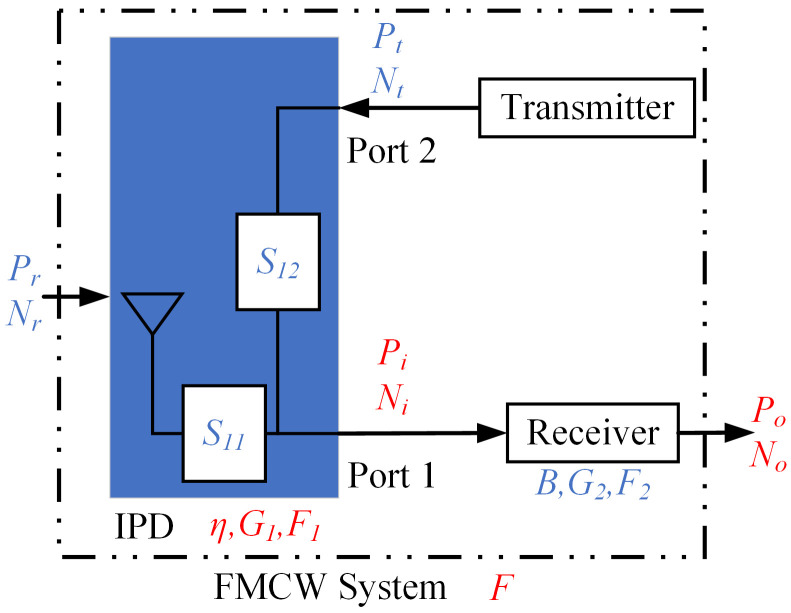
Simplified block diagram of the FMCW system with IPD structure.

IPD is the first structure when the radar signals entering the FMCW system, the two ports of which, respectively, connecting the transmitter and the receiver. Therefore, parameters such as isolation and return loss of IPD significantly affect the performance of the FMCW system, which makes optimizing the IPD structure a multi-objective optimization problem. The optimization generally aims for higher isolation and lower return loss. In practical applications, it is necessary to evaluate the IPD as a functional component of the FMCW system. This involves examining how the isolation and return loss values of the IPD affect the quality of the received signals. Developing a mathematical model is necessary for this analysis, which will help assess the priority of parameter optimization and provide theoretical guidance for optimizing the IPD structure. Figure 8 shows the simplified block diagram of the system with IPD, and the meaning of each parameter is summarized in Table 3.

The relationship between $P_{i}$ and $P_{r}$ is as follows:

(1) $$P_{i}=P_{r}\eta \tau ,$$

where τ is the ratio of the output power to the input power at port 1. We assume that the passive components of the system are lossless. Considering the isolation between port 1 and port 2, τ can be calculated by:

(2) $$\tau =1-{\left|S_{11}\right|}^{2}-{\left|S_{12}\right|}^{2}.$$

η in (1) is calculated as:

(3) $$\eta =\frac{P_{rec}}{P_{acc}},$$

where $P_{rec}$ is the power received by the antenna and $P_{acc}$ is the power output from IPD after considering the mismatch loss of the ports. η is related to the maximum gain $G_{0}$ of IPD:

(4) $$G_{0}=\eta D_{0},$$

where $D_{0}$ is the maximal directive gain value of IPD.

$N_{i}$ represents the noise power output from IPD and consists of three parts:

(5) $$N_{i}=N_{r}\eta \tau +N_{IPD}+N_{s},$$

where $N_{r}$ can be expressed as:

(6) $$N_{r}=kBT_{a},$$

where *k* is Boltzmann’s constant and takes the value of $1.3807\times 10^{-23}$ J/K. $T_{a}$ is the external noise temperature [29]. $N_{IPD}$ is the noise-equivalent temperature (NET) of IPD, which is expressed as:

(7) $$N_{IPD}=\left(1-\eta \right)\tau kBT_{0},$$

where $T_{0}$ is the standard reference temperature. $N_{s}$ is the value of noise leakage from the transmitter into the receiver, which is given by:

(8) $$N_{s}=N_{t}{\left|S_{12}\right|}^{2}.$$

The noise power output from the transmitter is as follows:

(9) $$N_{t}=N_{V}B,$$

where $N_{V}$ is the power spectrum of the transmitter output noise.

The gain $G_{1}$ and the noise factor $F_{1}$ can be expressed as:

(10) $$G_{1}=\frac{P_{i}}{P_{r}}=\eta \tau ,$$

(11) $$F_{1}=\frac{SNR_{r}}{SNR_{i}}=\frac{1}{\eta }+\frac{N_{V}{\left|S_{12}\right|}^{2}}{kT_{0}\eta \tau }.$$

Assuming that $NF_{2}$ is 6 dB, $N_{V}$ is −127 dBm/Hz, and $T_{a}$ = $T_{0}$ = 290 K. According to Friis formula [32], we get:

(12) $$F=F_{1}+\frac{F_{2}-1}{G_{1}}=\frac{\tau +4.9875\times 10^{4}{\left|S_{12}\right|}^{2}+2.981}{\eta \tau },$$

where τ, $S_{12}$, and η are functions of the discrete frequency points $f_{i}$. Based on (13), we calculate the weighted average value of *F* corresponding to each discrete frequency point $f_{i}$ in the range of $f_{0}\pm B/2$ and denote it by $Q_{d}$, which is called the average noise figure of the receiving system, and use it to evaluate the performance of IPD with different structural parameters:

(13) $$Q_{d}=10\mathrm{lg}\left(\frac{\left(w_{1}F_{f_{1}}+w_{N}F_{f_{N}}\right)\Delta f/2+\sum_{i=2}^{N-1}w_{i}F_{f_{i}}\Delta f}{B}\right).$$

The noise factor $F_{f_{i}}$ at the discrete frequency point $f_{i}$ can be calculated by (12). $w_{i}$ is the weight of the noise factor at each frequency point, which is calculated by (14). Δf is the frequency interval, *N* is the number of frequency points in the bandwidth, which is calculated by (15):

(14) $$w_{i}=1-\left|\frac{f_{i}-f_{0}}{B}\right|,$$

(15) $$N=\frac{B}{\Delta f}+1.$$

Thus, $Q_{d}$ of the IPD structure, calculated using empirical formulas in Section 2.3, is 42.86 dB in the frequency range of 8.45 GHz to 8.55 GHz, with a frequency interval of 0.01 GHz. This indicates poor performance. In the next chapter, we will use $Q_{d}$ as the optimization metric and apply specific optimization algorithms to improve this target.

## 4. Optimization for the Integrated Passive Device Structure

### 4.1. Optimization Strategy

In the optimization of microwave passive structures, finite element simulation software frequently features built-in optimization tools. While this method can effectively adjust design parameters, it often requires a significant amount of simulation time and imposes high performance demands on the computing system. Additionally, although the optimized parameters may have high accuracy, they may not align well with actual fabrication processes, particularly in complex cases involving integrated antennas and very fine microstrip line structures, such as the integrated passive device discussed in this paper. This often leads to time-consuming optimization processes with low accuracy.

**Figure 9:**
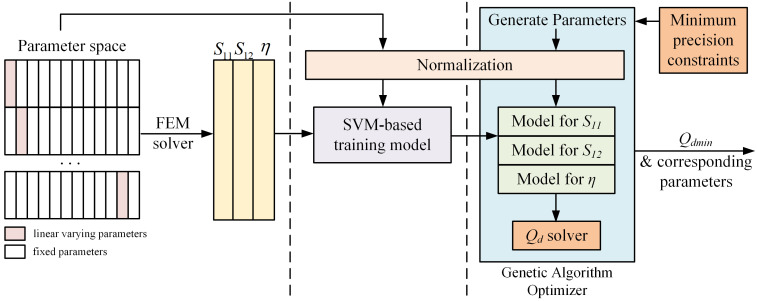
Optimization diagram.

To address the issue of time-consuming, some articles discuss the use of surrogate models to improve optimization efficiency [25,33,34,35,36]. We discovered that the method in [36] is especially effective for optimizing our model. The core idea is to establish surrogate models using support vector machine (SVM) for $S_{11}$, $S_{12}$, and efficiency η corresponding to different IPD structures and frequencies, thereby allowing for the calculation of the indicator $Q_{d}$. Subsequently, the genetic algorithm is employed with the objective of minimizing $Q_{d}$ to optimize the parameters of the IPD structure. We also introduce specific techniques, such as normalizing parameters across different scales and adding manufacturing constraints. This method can significantly reduce optimization time by using the surrogate models. The main process includes the establishment of the parameter space, model fitting using SVM, structure optimization, and evaluation of the optimization results, as illustrated in Figure 9. Each step will be discussed in detail in the following sections.

### 4.2. Process of the Optimization

To construct the surrogate model, we first need to establish a sample set for training, which involves defining the parameter space. It is essential to define the inputs and outputs clearly. According to Table 2, we identified 11 structural parameters as the input parameters for optimizing the IPD structure. Since our optimization objective is to minimize $Q_{d}$, which is a function of $S_{11}$, $S_{12}$, and efficiency η within a fixed segment as indicated in Section 3.2, we determine that there are three output parameters.

Next, we specify the boundaries and resolution for each input parameter. The parameters within the defined boundaries are varied linearly. We will conduct HFSS simulations at frequency points ranging from 8.45 GHz to 8.55 GHz with intervals of 0.01 GHz to obtain $S_{11}$, $S_{12}$, and efficiency η with different input structural parameters. Table 4 lists the boundary values and resolutions for each parameter, resulting in a total of 111 sample sets within the frequency band for subsequent training of the fitting model. In the last two columns of Table 4, fabrication accuracy indicates the minimum manufacturing precision of each input structural parameter, while the fixed values represent the constant settings of other parameters when the specified parameter is varied linearly.

From Table 4, it is evident that different parameters exhibit various dimensions and numerical ranges. We normalize these parameters using (16) to facilitate comparison on a standardized basis, where *x* represents the original data value, $x^{\prime }$ is the normalized data value, and min(X) and max(X) denote the minimum and maximum values of the dataset *X*, which represent the lower and upper bounds of the input structural parameters, respectively. By using this formula, the original data values can be scaled to the range of [−1,1]:

(16) $$x^{\prime }=\frac{2(x-\min(X))}{\max(X)-\min(X)}-1.$$

**Figure 10:**
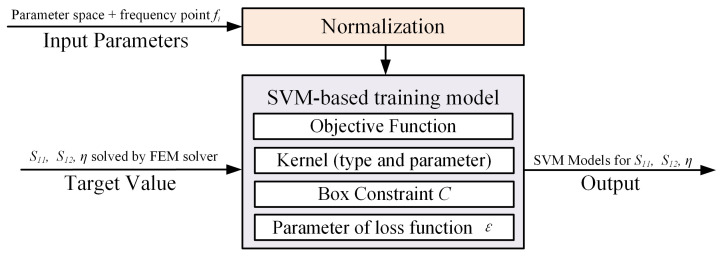
Illustration of SVM model training.

Support vector machine (SVM) is a supervised learning method used for solving classification and regression problems. As illustrated in Figure 10, when training the SVM model, we use the normalized parameter space along with the discrete frequency points $f_{i}$ as inputs, aiming to predict the values of $S_{11}$, $S_{12}$, and η. Given the initially small magnitudes of these target variables, they were scaled by a factor of 1000 to enhance numerical stability and improve the model’s training efficiency. After training, the predicted values from the SVM model were transformed back to their original scale by dividing them by 1000. The Mean Squared Error (MSE) serves as our optimization objective. Through multiple iterations of manual parameter tuning, we determined that the optimal kernel function type is the radial basis function (RBF), and we identified the related kernel parameter σ, and other key parameters, summarized in Table 5 and discussed in detail in [36].

**Figure 11:**
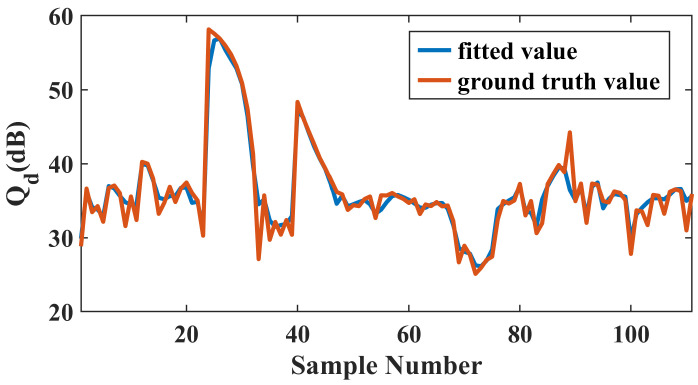
Comparison of the $Q_{d}$ computed from the support vector machine models fitting with the ground truth values.

We then calculate the values of $Q_{d}$ corresponding to both the finite element simulation and the fitting models, with the former serving as the ground truth for evaluation. Table 6 presents the mean squared errors of each parameter after fitting and Figure 11 illustrates the fitting curve of $Q_{d}$ within the sample space.

**Figure 12:**
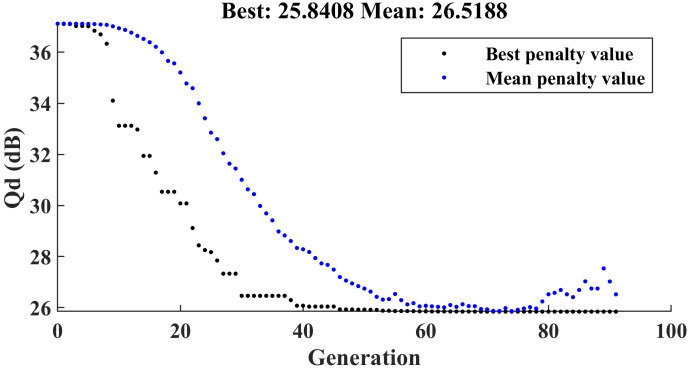
The iteration graph of the genetic algorithm optimization for $Q_{d}$.

We then use the genetic algorithm to find the minimum value of $Q_{d}$. First, we establish the upper and lower bounds for optimization. Additionally, we specify that during the iteration and mutation process of the genetic algorithm, any changes in the input structural parameters must be integer multiples of the fabrication accuracy values presented in Table 4. The normalized parameters are input into the support vector machine model, and the corresponding S-parameters and antenna efficiency are obtained. Based on (13), we calculate $Q_{d}$. Figure 12 shows the iteration graph of the genetic algorithm optimization. It can be observed that the optimal solution for $Q_{d}$ is 25.8408, which is significantly lower than the $Q_{d}$ obtained from the structural parameters calculated using the empirical formula. Table 7 records the optimal structural parameters selected by the genetic algorithm.

### 4.3. Analysis and Comparison of the Optimization Results

**Figure 13:**
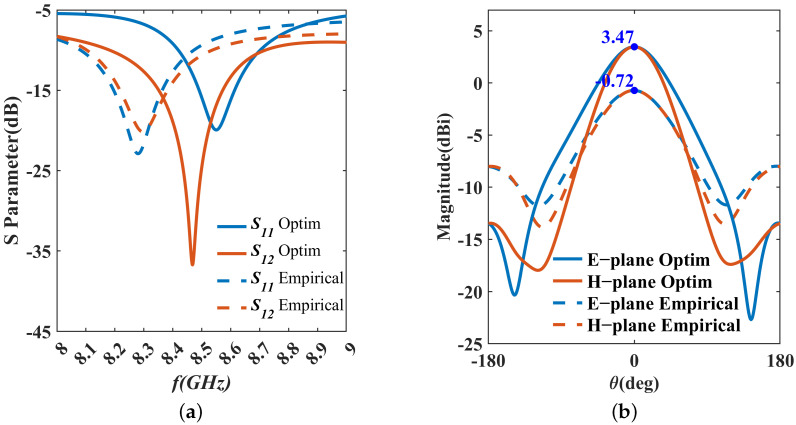
Results comparison of IPD optimized and designed by empirical formulas. (**a**) S-parameters; (**b**) E-plane and H-plane.

We input the optimized structural parameters into HFSS. Figure 13 shows the results of the S-parameters and gains.

Compared to the results derived from empirical formulas, we can see a significant improvement in the optimized S-parameters and gains. The simulated value of $Q_{d}$ is 27.65 dB, which represents a reduction of 15.21 dB compared to the empirical formula-based result of 42.86 dB. This demonstrates that the optimization strategy used in this paper for $Q_{d}$ is effective. Additionally, the single simulation time for the model in HFSS is 12 min, while the optimization algorithm requires only 1 millisecond for a single computation. This demonstrates that our optimization strategy significantly improves computation time with only a minor trade-off in accuracy.

## 5. Conclusions

This paper proposes an integrated passive device (IPD) structure based on the Fan-Out Wafer-Level Packaging (FOWLP) process, specifically applied to the RF front end of frequency-modulated continuous wave (FMCW) short-range radar systems. The objective is to enhance the vertical integration of FMCW front-end passive devices. In response to the excessive optimization metrics of the passive structure based on this process, which lack practical significance, and the necessity for theoretical analysis of the impact of FMCW RF front-end passive devices on receiver noise, we introduce a novel metric to evaluate the effect of the IPD structure on the average noise figure coefficient, $Q_{d}$, of the FMCW system’s receiver within the reception bandwidth. We present the derivation process, completing the mathematical modeling of how the IPD structure influences the introduction of environmental noise and transmitter noise into the receiver.

The IPD structure is initially designed using empirical formulas. However, upon assessing its performance, which yielded poor values, the optimization target is established to minimize $Q_{d}$. Support vector machines (SVM) are employed as a surrogate model, while genetic algorithms (GA) are utilized as the optimization method. This approach significantly enhances optimization efficiency. The optimized structural parameters are input into finite element simulation software for validation, and the results demonstrate that the optimized structure exhibits marked improvements in in-band isolation and a substantial reduction in the average noise figure $Q_{d}$, providing a theoretical foundation for practical fabrication and optimization.

## Figures and Tables

**Table 1:** Descriptions and Values of the structural parameters used in FOWLP.

Characters	Descriptions	Values
$\varepsilon_{r_{EMC}}$	permittivity of EMC	3.69
$H_{EMC}$	thickness of EMC substrate	400 µm
$\varepsilon_{r_{prep}}$	permittivity of prepreg	3.5
$H_{prep}$	thickness of the prepreg layer used as the substrate for the power divider	30 µm
$h_{prep}$	thickness of the other prepreg layers	10 µm
$D_{in}$	diameter of the through mold via	120 µm

**Table 2:** The parameters of IPD obtained by empirical formula-based design.

Parameters	$R_{\mathrm{out}}$	$W_{\mathrm{sub}}$	$W_{\mathrm{patch}}$	$L_{\mathrm{sub}}$	$L_{\mathrm{patch}}$	$X_{\mathrm{feed}}$	$W_{50}$	$W_{70}$	$L_{70}$	$R_{0}$	$L_{R_{0}}$
**Values (Units)**	297 µm	13.9 mm	11.5 mm	11.5 mm	9.1 mm	2.5 mm	68 µm	37 µm	5.4 mm	100 Ω	0 mm

**Table 3:** Descriptions of parameters used by FMCW RF system in Figure 8.

Characters	Descriptions
$P_{r}$	Echo signal power received by the antenna from space.
$N_{r}$	Noise power received by the antenna from space.
$P_{t}$	Signal power output from the transmitter.
$N_{t}$	Noise power output from the transmitter.
$S_{11}$	Voltage reflection coefficient at port 1 of IPD (connected to the receiver).
$S_{12}$	Voltage transmission coefficient from port 2 to port 1 of IPD.
η	Efficiency of IPD.
$G_{1}$	Gain of IPD.
$F_{1}$	Noise factor of IPD.
$P_{i}$	Output signal power of IPD (also input signal power of the receiver).
$N_{i}$	Output noise power of IPD (also input noise power of the receiver).
$P_{o}$	Output signal power of the receiver.
$N_{o}$	Output noise power of the receiver.
*B*	Bandwidth of the received signal.
$G_{2}$	Gain of the receiver.
$F_{2}$	Noise factor of the receiver.
*F*	Noise factor of the FMCW system.

**Table 4:** Values for parameter space construction.

Parameters	Units	Upper Boundary	Lower Boundary	Resolution	Fabrication Accuracy	Fixed Values
$R_{out}$	µm	400	200	25	1	284
$W_{sub}$	mm	35	20	2.5	0.01	31.7
$W_{patch}$	mm	20	10	1	0.01	15.7
$L_{sub}$	mm	35	20	2.5	0.01	24.75
$L_{patch}$	mm	9	7	0.2	0.01	8.85
$X_{feed}$	mm	3	1	0.2	0.001	2.41
$W_{50}$	µm	100	40	5	1	66
$W_{70}$	µm	50	20	5	1	36
$L_{70}$	mm	3.74	6.88	0.314	0.001	1.51
$R_{0}$	Ω	150	50	10	1	61
$L_{R_{0}}$	mm	1.2	0	0.1	0.01	0.17

**Table 5:** Parameter settings for SVM fitting models.

Model	Kernel Type	RBF Kernel Parameter σ	Box Constraint C	Loss Funciton Parameter ϵ	Objective Function
$S_{11}$	RBF	0.53	74.17	7.42	MSE
$S_{12}$	RBF	0.42	86.44	8.64	MSE
η	RBF	0.53	26.78	2.68	MSE

**Table 6:** Values of the mean squared error of the fitted parameters and $Q_{d}$.

Parameters	$S_{11}$	$S_{12}$	η	$Q_{d}\;$(dB)
**Mean squared error**	0.0008	0.0007	0.0004	2.4176

**Table 7:** The parameters of IPD obtained by the genetic algorithm optimization.

Parameters	$R_{\mathrm{out}}$	$W_{\mathrm{sub}}$	$W_{\mathrm{patch}}$	$L_{\mathrm{sub}}$	$L_{\mathrm{patch}}$	$X_{\mathrm{feed}}$	$W_{50}$	$W_{70}$	$L_{70}$	$R_{0}$	$L_{R_{0}}$
**Values**	288 µm	31.17 mm	15.21 mm	23.69 mm	8.82 mm	2.472 mm	66 µm	39 µm	5.432 mm	62 Ω	0.14 mm

## Data Availability

Data are contained within the article.
